# Research progress of the paraventricular thalamus in the regulation of sleep–wake and emotional behaviors

**DOI:** 10.1002/ibra.12034

**Published:** 2022-04-30

**Authors:** Xiao‐Li Bu, Cheng‐Xi Liu, Bao Fu

**Affiliations:** ^1^ Department of Intensive Care Medicine Affiliated Hospital of Zunyi Medical University Zunyi Guizhou China; ^2^ Guizhou Key Laboratory of Anesthesia and Organ Protection Zunyi Medical University Zunyi Guizhou China

**Keywords:** emotional behaviors, paraventricular thalamus, sleep, wakefulness

## Abstract

The paraventricular thalamus (PVT) is a major component of the midline structure of the thalamus. It is one of the nonspecific nuclei of the thalamus, which plays a great role in the regulation of cortical arousal. PVT, an important node in the central nervous system, sends widespread outputs to many brain regions and also accepts plentiful inputs from many brain regions to modulate diverse functions, including sleep–wake state, attention, memory, and pain. Recently, with the increasing prevalence of sleep disorders and mood disorders, people pay great attention to PVT, which was implicated in arousal and emotional behaviors. Therefore, the main purpose of this review is to illustrate the characteristic of PVT to provide a reference for future research.

## Graphical abstract

Paraventricular thalamus (PVT) is a key node in the brain neural network, which is involved in the regulation of sleep–wake and emotional behaviors, such as reward, anxiety, fear, stress, and so on. The corresponding neurobiological functions and neural circuits of PVT are shown in the figure.

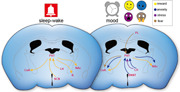

## 1 INTRODUCTION

The importance of the thalamus for arousal has been recognized since the reticular formation theory was proposed first.^1^ The thalamus, an integral component of the thalamus–cortex and cortical–cortical arousal circuits, is the gateway to the regulation of cortical arousal.^2^ It can be divided into specific and nonspecific thalamus according to its projections, with the paraventricular thalamus (PVT) being a critical component of the nonspecific thalamus and playing a key role in maintaining cortical arousal and emotional behaviors.^3^

A growing body of literature has demonstrated that the PVT is involved in the regulation of numerous physiological and pathological functions, such as drug addiction, pain regulation, and learning memory.^4^, ^5^, ^6^ With the increasing prevalence of wakefulness disorders and neuropsychiatric diseases, the PVT is of extreme interest in the regulation of sleep–wake and emotional behaviors. Therefore, this paper considers the anatomical structure, electrophysiological characteristics, neural network, and physiological functions of PVT, with a view to providing references for subsequent studies.

## 2 ANATOMY AND NEURONS OF PVT

PVT is one of the midline nuclei of the thalamus, located in the subventricular canal of the third ventricle; it is divided into an anterior and a posterior part in the rostrocaudal axis and is continuous with the subthalamic paraventricular system anteriorly and with the periventricular gray matter of the midbrain aqueduct posteriorly. PVT neurons are mainly glutamatergic neurons that synthesize and release glutamatergic neurotransmitters and are involved in the regulation of various neurological functions.^7^, ^8^ The release of glutamate requires synaptic vesicles, and the entry of synthetic glutamate into the vesicles requires the involvement of specific glutamate transporters (VGLUTs).^9^, ^10^ Previous studies found that only VGLUT2 messenger RNA‐positive neurons were observed in the PVT by in situ molecular hybridizations, suggesting that glutamatergic neurons in the PVT are of the VGLUT2 type.^5^ Like other dorsal thalamic nuclei, PVT neurons do not express the inhibitory γ‐aminobutyric acid (GABA) and the fast‐issuing interneuron marker parvalbumin (PV).^11^ PVT neurons in rodents, monkeys, and humans highly express calretinin (CR), and the vast majority of CR+ neurons coexpress the glutamatergic neuronal marker Vglut2, but only 61.52 ± 2.62% of Vglut2+ neurons express CR, suggesting subpopulation heterogeneity within PVT neurons, and that CR could serve as a unique marker for PVT neurons.^12^, ^13^, ^14^ PVT neurons also express 28 kDa calcium‐binding protein and a variety of neurotransmitter receptors, such as appetaminergic receptors, melatonergic receptors, and dopamine receptors, among which PVT neurons are characterized by dopamine D2 receptors (D2Rs).^15^, ^16^, ^17^, ^18^ D2Rs are predominant on PVT neurons, and D2R+ and D2R− neurons are distributed in a gradient along the anterior–posterior axis of PVT, and these two types of neurons have different neurobiological functions in sleep–wake and emotional cognitive behaviors.^18^

## 3 ELECTROPHYSIOLOGICAL CHARACTERISTICS OF THE PVT

PVT neurons have an oblong or multipolar cell body with a long‐axis diameter of 12–30 μm and 3–7 dendrites emanating from the cell body and extending hundreds of micrometers in different directions.^19^ In addition, PVT neuronal protrusions also emit dendritic branches and extend to the dorsal third ventricle, forming a unique connection with the ventricular tegmental cell layer.^20^ The resting membrane potential of PVT neurons is maintained by plentiful ion channels, including inwardly rectifying potassium channels, hyperpolarization‐activated nonselective cation and twik‐related acid‐sensitive K+ channel, and firing patterns are regulated by high‐voltage activated and low‐voltage‐activated Ca^2+^ channels.^21^ PVT neurons exhibit two different firing patterns: tonic firing with only a single action potential and burst firing containing more than two action potentials.^22^, ^23^ Firing patterns of PVT neurons correlate with arousal state and affect their responsiveness to excitatory synaptic inputs. For example, burst firing is seen in pathological states, such as slow‐wave sleep and epilepsy. However, tonic firing is mostly seen in the waking state.^22^ Using the membrane clamp technique, it was demonstrated that PVT neurons in isolated brain slices have the above arousal state‐dependent action potential firing pattern.^24^ More importantly, the frequency of action potential and intrinsic electrophysiological characteristics of PVT neurons have arousal‐related circadian rhythmic changes. A recent study has shown that a higher frequency of PVT neuron firing in acutely isolated brain slices of PVT prepared during the active phase, including spontaneous burst and tonic firing and a relatively depolarized (−64 ± 1 mV) membrane potential level. In contrast, the resting membrane potential of PVT neurons was in a relatively hyperpolarized state (−70 ± 1 mV), with less spontaneous action potential generation. Considered together, PVT neurons in the waking state are dominated by tonic firing, while they are dominated by burst firing in the sleeping state.

Emotional behaviors depend on a good state of arousal, so the electrophysiological changes of PVT neurons involved in emotional cognitive behaviors are the same as the electrophysiological properties of PVT neurons during the circadian cycle. It was shown that the majority of D2R+ neurons in the anterior–posterior PVT axis without electroshock stimulation were resting in mice, and most D2R neurons showed tonic or burst firing.^18^ In contrast, the proportion of D2R+ neurons in the anterior–posterior PVT axis that was spontaneously firing increased, and the proportion of D2R− neurons that was spontaneously firing decreased in mice with an electric shock to the plantar area. That is, the activity of the D2R– neuron population in the anterior PVT (aPVT) decreased when aversive stimulation was given, while the activity of D2R+ neurons in the posterior PVT (pPVT) increased.

## 4 ANATOMICAL PROJECTIONS OF THE PVT

**Figure 1:**
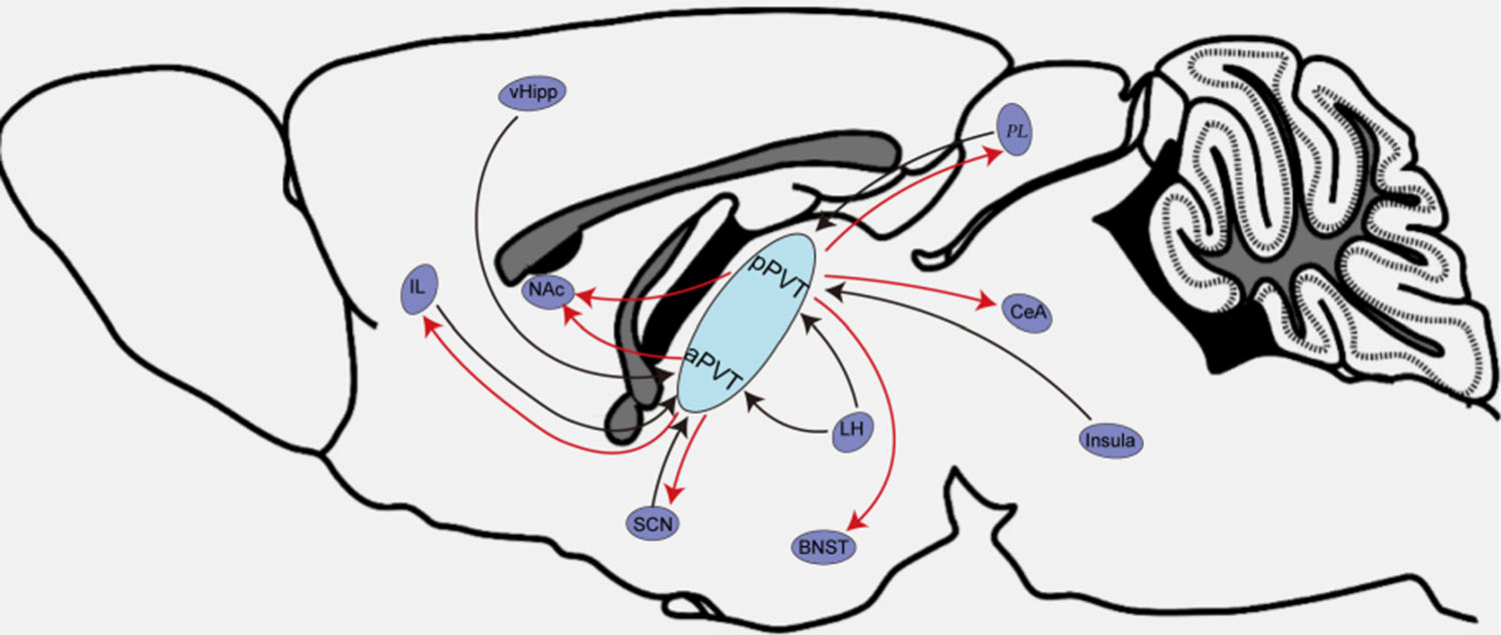
Schematic diagram of the neural network of the paraventricular thalamus. aPVT, anterior paraventricular thalamus; BNST, nucleus of the bed of the terminal striatum; CeA, central amygdala; IL, inferior limbic cortex; LH, lateral hypothalamus; NAc, nucleus ambiguus; PL, prelimbic cortex; pPVT, posterior paraventricular thalamus; SCN, supraoptic nucleus; vHipp, ventral hippocampus. [Color figure can be viewed at wileyonlinelibrary.com]

The PVT is divided into anterior and posterior parts according to its anatomical location, where there are significant differences in the input–output nerve fibers and nerve functions concerning the anterior and posterior parts. aPVT mainly receives fiber projections from the ventral hippocampal subiculum and the infralimbic cortex (IL) associated with arousal. Conversely, pPVT mainly receives fiber projections from the prelimbic cortex (PL), IL, and anterior margin of the insula, which mainly transmits information, such as taste and visceral sensation.^2^, ^25^ PVT receives dense hypocretin (Hcrt) neuronal projections from the lateral hypothalamus (LH), with pPVT receiving denser Hcrt neuronal fiber projections than aPVT.^26^ aPVT projects to a wide range of limbic areas, mainly to the suprachiasmatic nucleus, which is involved in circadian rhythms. In contrast, pPVT projects to a more restricted area, mainly to the bed nucleus of the stria terminalis (BNST) and the central nucleus of the amygdala (CeA), which mediates the regulation of anxiety and fear.^27^, ^28^, ^29^, ^30^ In addition, aPVT sends more projections to the dorsal medial part of the nucleus accumbens (NAc) shell, mediating appetite‐related behaviors. However, pPVT sends more projections to the ventral medial part of the NAc shell, mediating aversion‐related behaviors.^27^ Furthermore, it was found that aPVT has fiber projections to pPVT, but no projections from pPVT to aPVT were found, suggesting that information transfer within the PVT may be unidirectional.^30^ In summary, these anatomical connections suggest that the PVT plays an important role in coordinating and modulating arousal and affective cognitive behaviors, with aPVT playing a dominant role in arousal modulation and pPVT being primarily involved in affective cognitive modulation. Neural network of the PVT is shown in Figure 1.

## 5 ROLE OF PVT IN THE REGULATION OF SLEEP–WAKE

Arousal is the basis of activities, such as sensory, motor, thinking, and consciousness. Poor arousal can lead to reduced alertness, inattention, and reduced cognitive function. Patients with paramedian thalamic injury exhibit drowsiness as PVT is a major component of the paramedian thalamus and shows significant changes in neuronal activity during circadian rhythms, and is an important component of the upstream arousal pathway as well.^31^ It has been shown that chemogenetic inhibition of PVT glutamatergic neurons was found to significantly reduce the time of arousal, cause arousal fragmentation, and make arousal unsustainable. Destruction of PVT glutamatergic neurons with DTA caused persistent arousal impairment, as evidenced by reduced arousal duration, fragmentation, and increased electroencephalography delta power.^32^ This study further found that activation of PVT glutamatergic neurons during sleep induced a rapid transition from sleep to wakefulness, while activation of PVT during anesthesia increased cortical excitability and shortened the time required for the transition from anesthesia to wakefulness. In addition, it was further found that mice exhibited coma and lethargy using the electrical destruction technique.^33^

PVT is also involved in the regulation of arousal at the level of neuronal subtypes. Studies have shown that varying degrees of optogenetic activation of CR+ neurons in the dorsal thalamus induce sleep‐to‐wake transition, active movement, microarousal, and a brief interruption of sleep, respectively.^34^ Similarly, optogenetic activation of PVT CR+ neurons promotes arousal, and it was further demonstrated that PVT CR+ neurons are involved in regulating starvation‐induced arousal.^13^ These studies suggest that PVT not only regulates arousal in physiological states but is also involved in maintaining arousal in different behavioral states (starvation). Similarly, it was shown that D2R+ and D2R− neurons distributed along a gradient on the rostrocaudal axis of PVT, with the former mainly confined to pPVT and the latter distributed in aPVT.^18^ This study shows that D2R+ neurons at pPVT mediate mainly the regulation of emotional behavior, while D2R− neurons at aPVT mediate mainly the regulation of sleep–wake. The study has shown that optogenetic activation of D2R− PVT neurons projecting to the submarginal cortex abrogate the inhibitory effect of significant stimuli on this pathway, thereby altering cortical arousal status. Chemogenetic activation of D2R− PVT neurons showed reduced wakefulness and increased nonrapid eye movement (NREM) sleep. This result is consistent with previous studies that activation of aPVT increases NREM sleep time and decreases wakefulness time.^35^ In conclusion, the above results demonstrate that the activity of D2R− PVT neurons is negatively correlated with arousal. In summary, PVT is involved in the regulation of sleep–wake as a whole, and its regulatory effects should be specific to a particular neuronal subtype. Therefore, the identification of neuronal subtypes in PVT is a problem that needs to be solved.

The main neural circuits with which PVT exerts its arousal‐promoting effect have been identified as the LH–PVT–NAc. It has been shown that activation of the PVT–NAc pathway induces a switch from sleep to wakefulness.^32^ Conversely, inhibition of this pathway reduces the level of arousal. It has been shown that PVT glutamatergic neurons receive innervation from Hcrt neurons located in the LH and chemogenetic inhibition of Hcrt neuronal input in the PVT significantly reduces arousal. Optogenetic activation of the LH_Hcrt_–PVT pathway induces a switch from sleep to wakefulness. Locus coeruleus (LC)–PVT pathway is another neural pathway for the arousal‐promoting effect of PVT. LC is involved in the regulation of arousal, cognition, and attention and sends projections to PVT, which regulates the activity of PVT neurons by modulating the inhibitory inputs to PVT, thus putting the body in a state of alertness.^36^ In addition, it has been demonstrated that optogenetic activation of TH:LC–PVT terminals promotes arousal from isoflurane anesthesia, and chemogenetic inhibition of TH:LC–PVT terminals delays emergence from anesthesia in mice.^37^

On the other hand, PVT is another important target for melatonin to exert its sleep‐promoting effects. Recently, it has been demonstrated using the membrane clamp technique that melatonin specifically acts on melatonin receptor 1 on the PVT, inhibiting PVT neuronal excitability by enhancing cytosolic delayed rectifier potassium currents. Furthermore, melatonin inhibits excitatory inputs to PVT glutamatergic neurons, which, in turn, reduces PVT firing. In addition, local administration of melatonin in PVT significantly reduces the time of wakefulness, increases the duration of NREM sleep and REM sleep, and facilitates the transition from wakefulness to sleep.^32^

In summary, PVT exerts its arousal‐promoting effects via the LH–PVT–NAc and LC–PVT pathways, while melatonin inhibition of PVT activity produces sleep‐promoting effects. However, the neural pathways of PVT and its additional neuronal subtypes need to be further explored to clarify its broad neurobiological functions.

## 6 ROLE OF THE PVT IN EMOTIONAL BEHAVIORS

Neuropsychiatric disorders have received considerable attention in recent years, such as depression, anxiety, stress, and so on. It has been found that symptoms of mood disorders are often accompanied by various abnormal changes in the thalamus, such as reduction in thalamic volume and loss of neuronal function.^38^ PVT, an important part of the thalamus, plays a great role in emotional cognition, which will be described in the following aspects.

### 6.1 Reward

It has been shown that when electrodes are placed in the PVT, rats are found to continuously touch the switch button to give current stimulation to achieve intracranial self‐stimulation (ICSS).^39^ Similarly, using optogenetic methods to activate the PVT–NAc projections, mice exhibit an increase in the number of pressing to obtain light stimulation to achieve intracranial stimulation, suggesting that stimulation of the PVT–NAc circuit can reinforce operant behavior.^40^ Furthermore, using two‐photon calcium imaging, it was found that the activity of PVT neurons projecting to the NAc was reduced when reward‐related cue stimuli were given to mice and the neuronal activity was modulated by inputs from the prefrontal cortex and LH.^41^ The above studies suggest that PVT plays an important role in rewarding behaviors.

The PVT is involved in the modulation of rewarding behavior as a whole, but its specific subregions exert distinct effects in this process. One study reported that ICSS is achieved by placing stimulating electrodes between the LH and the medial forebrain, with stimulation thresholds showing a dose‐dependent decrease with the injection of inhibitory neuropeptides (cocaine and amphetamine) at aPVT, whereas the injection of an appetite antagonist reverses this effect, demonstrating that inhibition of aPVT neuronal activity promotes rewarding behavior.^42^ Similarly, activation of aPVT–NAc and aPVT–CeA projections using optogenetic methods reveals that mice spend less time in the preferred location.^43^ Cue‐induced sucrose magazine entries are unaffected by chemogenetic inhibition of both anterior and posterior rat PVT,^44^ and cue‐induced lever pressing for a sucrose reward is unaffected by pharmacological inhibition of the rat posterior PVT via microinjection of the GABA agonist muscimol.^43^ Pharmacological suppression of the rat anterior PVT, on the other hand, increases cue‐induced lever‐pressing for sucrose rewarding. To summarize, PVT activation has a broad effect on reward promotion and aPVT plays a key part in this process in the appropriate subregion, whereas pPVT activation has little effect on reward behavior. Instead, pPVT plays a dominant role in the perception of reward‐related stimuli. A study using fiber‐optic calcium signaling showed that the activity of pPVT neurons was reduced when mice were given rewarding stimuli, including access to a female conspecific (for males) or thermoneutral zone. However, pPVT neuronal activity was increased when aversive stimuli (electric shock or tail suspension) were given. aPVT neuronal activity was always inhibited when either rewarding or aversive stimuli were given.^18^ The study also used the ex vivo membrane clamp technique to discover that most D2R + PVT cells (primarily in the pPVT) were silent, while many D2R − PVT cells (primarily in the aPVT) exhibited tonic firing and burst firing in mice not given foot shock in advance, whereas mice given foot shock in advance had a significantly increased proportion of D2R + PVT cells firing, while a decrease in the proportion of D2R – PVT cell. In summary, aPVT is engaged in the control of rewarding behavior and pPVT neurons can respond to stimuli more quickly than aPVT in response to rewarding or unpleasant stimuli, likely conveying this neural signal to the aPVT, which then regulates reward‐related behavior. When rewarding or aversive stimuli were given, however, aPVT neuronal activity was always inhibited, possibly because neurons in the aPVT were not uniformly activated, as single‐cell membrane clamp recordings revealed that when mice were given conditioned rewards, aPVT neuronal firing frequency increased or decreased.^43^

### 6.2 Depression

Recent studies have shown that PVT is also involved in the regulation of depression‐like behaviors. It was found that when mitochondrial DNA was absent in PVT neurons or when mitochondrial dysfunction was present, mice exhibited a depression‐like state: mainly in the form of reduced turn‐times, increased corticosterone levels, increased sleep, and increased food intake. Similarly, patients with a mitochondrial disease with mood disorders have similar lesions in their postmortem brains.^45^ From the above, it is clear that defects in mitochondrial function of PVT neurons are an important cause of the onset of depression. Mice can show depression‐like symptoms by injecting tetanus toxin at the PVT to inhibit PVT neuronal activity.^45^ Interestingly, in a recent study, the PVT was found to play an opposite role in the regulation of depressive behaviors. In this study, tetanus toxin was used to inhibit PVT neuronal activity, and mice showed reduced resting time in a forced swimming test. However, long‐term chemogenetic activation of PVT neurons resulted in reduced activity in mice.^46^ It is clear from the above that PVT neuronal activity is involved in the regulation of depressive behaviors, but the direction, subregions, and pathways that produce these effects remain to be investigated.

### 6.3 Anxiety

It was demonstrated that optogenetic activation of the PVT–CeA pathway resulted in a decrease in the residence time of mice in the open arm of the elevated cross‐maze test, while the opposite behavioral effect was produced by optogenetic inhibition of this circuit.^47^, ^48^ It follows that activation of the PVT–CeA projection promotes anxiety‐like behaviors. In one study, appetite peptide (Hcrt) was injected at pPVT by microinjection and mice showed reduced residence time in the open arm and reduced entry into the open arm in the elevated cross maze test.^49^, ^50^ In contrast, mice injected with Hcrt antagonists at pPVT showed rapid access to social areas in socialization tests.^51^ On the other hand, mice injected with a GABA receptor agonist (muscimol) at pPVT showed reduced time in the open arm and reduced entry into the open arm in the elevated cross maze test.^52^ Furthermore, it has been reported that neither injection of GABA receptor inhibitors at the aPVT nor optogenetic activation of aPVT–NAc circuit affected the time in the open arm and the number of entries into the open arm in the elevated cross‐maze test in mice.^52^, ^53^ Taken together, it is clear that PVT mediates the regulation of anxiety‐like behavior in which pPVT plays a dominant role.

### 6.4 Stress

It has been shown that the level of c‐Fos expression in PVT neurons is increased after alcohol withdrawal in mice, which was also demonstrated in rats.^54^, ^55^ Similarly, the level of c‐Fos expression in PVT neurons was found to be increased in mice after electroshock on their feet.^48^ The level of c‐fos expression in PVT neurons is also increased after subjecting rats to a forced swim test.^56^ In addition, it was found that the calcium signals of both aPVT and pPVT were increased by an electric shock to the foot of rats.^44^ One study found that the c‐Fos expression level of pPVT increased more than aPVT in rats subjected to injurious stimuli, such as electric shock and tail suspension.^57^ In summary, PVT is involved in the stress process and pPVT plays a major role.

## 7 CONCLUSIONS AND PROSPECTS

PVT is a critical node for a variety of neural functions and plays a significant role not only in sleep and wakefulness maintenance but also in emotion regulation, cognition, and memory. However, our understanding of PVT is limited, so it is far‐reaching to explore the neuronal types of PVT, the inputs and outputs of PVT, receptors on PVT neurons, and potential mechanisms of transmitter effects, which can provide more therapeutic strategies for patients with clinical coma, sleep disorders, and emotional abnormalities.

## AUTHOR CONTRIBUTIONS

Cheng‐Xi Liu collected data, Xiao‐Li Bu contributed to drafting, and Chengxi Liu and Bao Fu supervised the work. All authors have read and agreed to the published version of the manuscript.

## CONFLICTS OF INTEREST

The authors declare no conflicts of interest.

## ETHICS STATEMENT

Not applicable.

## Data Availability

Data sharing not applicable to this article as no datasets were generated or analyzed during the current study.
